# Few-Shot Segmentation of 3D Point Clouds Under Real-World Distributional Shifts in Railroad Infrastructure

**DOI:** 10.3390/s25041072

**Published:** 2025-02-11

**Authors:** Abdur R. Fayjie, Mathijs Lens, Patrick Vandewalle

**Affiliations:** EAVISE, ESAT-PSI, KU Leuven, 3000 Leuven, Belgium; mathijs.lens@kuleuven.be (M.L.); patrick.vandewalle@kuleuven.be (P.V.)

**Keywords:** railway monitoring, distributional shifts, few-shot learning, point cloud segmentation

## Abstract

Industrial railway monitoring systems require precise understanding of 3D scenes, typically achieved using deep learning models for 3D point cloud segmentation. However, real-world applications demand these models to rapidly adapt to infrastructure upgrades and diverse environmental conditions across regions. Conventional deep learning models, which rely on large-scale annotated datasets for training and are evaluated on test sets that are drawn independently and identically from the training distribution, often fail to account for such real-world changes, leading to overestimated model performance. Recent advancements in few-shot learning, which aim to develop generalizable models with minimal annotations, have shown promise. Motivated by this potential, the paper investigates the application of few-shot learning to railway monitoring by formalizing three types of distributional shifts that are commonly encountered in such systems: (a) in-domain shifts caused by sensor noise, (b) in-domain out-of-distribution shifts arising from infrastructure changes, and (c) cross-domain out-of-distribution shifts driven by geographical variations. A systematic evaluation of few-shot learning’s adaptability to these shifts is conducted using three performance metrics and a predictive uncertainty estimation metric. Extensive experimentation demonstrates that few-shot learning outperforms fine-tuning and maintains strong generalization under in-domain shifts with only ~1% performance deviation. However, it experiences a significant drop in performance under both in-domain and cross-domain out-of-distribution shifts, pronounced when dealing with previously unseen infrastructure classes. Additionally, we show that incorporating predictive uncertainty estimation enhances few-shot learning applicability by quantifying the model’s sensitivity to distributional shifts, offering valuable insights into the model’s reliability for safety-critical applications.

## 1. Introduction

Railways are among the busiest modes of global transportation and are essential for efficiently moving people, freight, and containers across regions. They frequently require rapid and consistent maintenance to ensure uninterrupted operations, resulting in substantial maintenance costs, time investment, and reliance on manual labor [1,2,3]. Automated monitoring systems can address these challenges by offering a viable and rapid solution while also reducing the reliance on manual labor and the associated costs. Such systems necessitate an accurate understanding of 3D scenes, which is typically achieved by utilizing deep learning models for point cloud segmentation [4,5]—a task that involves classifying each point into its corresponding category, thereby dividing the point cloud into semantically meaningful regions. Most of these models are constrained by the requirement of large-scale annotated data for training. Creating such annotations for railway point cloud data is particularly challenging as it involves cumbersome manual labor and is time-consuming, expensive, and error-prone due to the intricate geometrical structures of the objects involved. Furthermore, their performance is typically evaluated on a test set that is identically and independently distributed (i.i.d.) relative to the training set, often causing overestimation of their actual effectiveness.

The i.i.d. assumption is often violated in real-world applications, potentially leading to catastrophic outcomes in safety-critical systems such as railway monitoring, where accuracy in decision-making is of paramount importance. Gawlikowski et al. [6] attributed the failure of deep learning models in real-world deployment for safety-critical systems to their inability to distinguish between in-domain (ID) and out-of-distribution (OOD) samples, their sensitivity to distributional shifts, and their lack of reliable uncertainty estimation. Railway monitoring presents a complex and dynamic landscape, often resulting in distributional shifts. For example, maintenance activities can introduce new types of infrastructure as part of an upgrade process, leading to the emergence of novel classes not encountered during the model’s training phase. Additionally, timely detection and removal of vegetation are crucial for ensuring smooth operations, but vegetation characteristics vary significantly across regions. Furthermore, railway environments differ substantially based on geographical location, encompassing variations in infrastructure types, weather conditions, and environmental settings, such as rural versus urban areas. These scenarios, characterized by significant variations between training and test conditions [7,8,9], are referred to as OOD scenarios. A model designed for railway monitoring is expected to generalize effectively under such OOD conditions. However, in practice, this often results in unreliable predictions [6,10]. Consequently, assessing a model’s generalization capability—both in terms of performance and uncertainty in its decisions—becomes a cornerstone for model selection and their reliable deployment.

The scarcity of large-scale point-wise annotations for 3D point clouds and the critical need for effective generalization motivate this paper exploring few-shot learning (FSL) [11,12,13]—a relatively new deep learning paradigm that aims to develop models that are capable of generalizing to unseen novel classes using only a minimal amount of labeled data. In recent years, FSL has demonstrated significant potential in the image and text domains [14,15]. However, its application to 3D point clouds remains relatively limited. Although some works have explored FSL for point cloud segmentation in indoor environments [16,17,18,19,20,21,22,23,24,25,26,27], its use in railway environments is still largely under-explored. In contrast to controlled indoor environments with generally dense point clouds, railway environments present far more challenging scenarios: inherent environmental and sensor noises, unintentional occlusions, variations in lighting conditions, and significant variations in geometric shapes within the same class (e.g., vegetation). Long-range LiDAR scanners used for railway monitoring often collect data over extended distances, resulting in sparse points in many regions within the point cloud compared to the shorter-range detailed high-resolution scanning in indoor environments. Additionally, the railway environment has fewer features, but these features are much larger and more spread out compared to the smaller, more numerous objects found indoors.

This paper investigates the generalization capability of FSL under real-world distributional shifts encountered in railway monitoring systems, contributing to a deeper understanding of model effectiveness in decision-making for safety-critical applications. To achieve this, we formalize three types of distributional shifts: (a) ID shift, (b) in-domain OOD shift, and (c) cross-domain OOD shift, further detailed in Section 2.2. A systematic evaluation is conducted using three performance metrics, along with a predictive uncertainty estimation metric to assess model sensitivity by quantifying prediction uncertainties. As demonstrated through experimental validation, this evaluation provides valuable insights into model development, selection, and deployment for reliable railway monitoring systems. Although prior studies have separately explored distributional shifts and uncertainty estimation in point cloud segmentation, to the best of our knowledge, this is the first to comprehensively evaluate few-shot segmentation for railroad monitoring under real-world distributional shifts.

The structure of the paper is as follows: Section 2 presents a comprehensive review of the related works, providing background, context, and the problem setup. Section 3 outlines the materials and methods used in the study. In Section 4, we detail the experimental setup. Section 5 delves into an in-depth analysis of the results, examining the key findings. Section 6 offers a discussion of the implications and relevance of the study’s outcomes. In Section 8, the conclusion is presented.

## 2. Background

### 2.1. Related Work

**Three-dimensional point cloud segmentation for railroad infrastructure.** Earlier works focused on developing heuristic-based methods [28,29,30,31,32,33] to detect railway infrastructure by exploiting underlying geometrical or morphological patterns in 3D point clouds.  Sánchez-Rodríguez et al. [34] integrated heuristics with an SVM classifier—a classical machine learning technique to detect rail tracks, cantilever arms, power line wires, and lining in railway tunnels. These methods are based on simple predefined rules derived from domain knowledge, making them computationally efficient and requiring minimal data. However, they heavily rely on handcrafted features designed by human experts, which restricts their application to simple detection tasks, typically involving regular, well-defined linear or circular objects. Furthermore, their adaptability is limited as they struggle to handle variations such as environmental changes, outliers, scaling, or noise.

The advancements in deep learning for point cloud segmentation prompted Soilán et al. [35] to explore two well-known point cloud segmentation networks, PointNet [36] and KP-Conv [37], for railway tunnel detection, achieving performance on par with the heuristic-based approach proposed by Sánchez-Rodríguez et al. [34]. PointNet, a pioneering work by Qi et al. [36], is the first network that directly inputs unstructured point clouds into a neural network, learning per-point features through shared multi-layer perceptrons and pooling functions. KP-Conv introduces a flexible and deformable convolutional operator that applies point convolutions to kernel points and their neighbors in Euclidean space. Chen et al. [38] applied a multi-scale Hierarchical Conditional Random Field (HiCRF) model to classify electrification assets in railway infrastructure by capturing spatial relationships. FarNet [39] introduced an attention module to aggregate spatial attention into feature information, learning from the spherical projection of point clouds. Ton et al. [40] adopted PointNet++ [41], SuperPoint Graph [42], and Point Transformer [43] for the semantic segmentation of railway infrastructure in catenary arches. PointNet++ extends PointNet by incorporating a grouping function to capture richer feature information, while SuperPoint Graph leverages object contextual relationships to partition point clouds into superpoint graphs, with each superpoint embedded via PointNet and processed by a graph convolutional network. Point Transformer incorporates self-attention layers for enhanced feature learning. However, it is computationally intensive, as is SuperPoint Graph. Kharroubi et al. [44] used KP-Conv, LightGBM, and Random Forest for semantic segmentation in railway environments. These models, trained in a supervised manner, address the limitations of heuristic-based approaches by automatically extracting meaningful features from data, enabling their application to complex high-dimensional railway segmentation problems, albeit with higher computational complexity and a significant requirement for labeled data. However, as typically evaluated on a test set drawn i.i.d. from the same distribution as the training data, these models do not account for the real-world variations encountered in railway environments, often resulting in an overestimation of model performance. Additionally, these works overlook uncertainty quantification in the model’s predictions, which is crucial for accurate decision-making, especially in identifying highly uncertain samples for further review by human counterparts.

**Distributional shifts and OOD conditions in point clouds.** Distributional shifts in point clouds can be categorized into ID shifts and OOD shifts. ID shifts arise from minor variations in the test set relative to the training set, characterized by positional, rotational, and scaling disturbances, along with jitter, outliers, sparsity, and corruptions. TriangleNet [45] addresses these shifts in point cloud classification by utilizing arbitrary SO(3) rotations, demonstrating high performance even with sparse point clouds using as few as 16 points sampled for each object. PointASNL [46] introduces adaptive sampling and a local–nonlocal module to handle inherent noise and outliers in point clouds. RobustPointSet [47] proposes a benchmark for evaluating point cloud classifiers on transformations, such as noise, rotation, sparsity, and translation. DUP-Net [48] and PointGuard [49] leverage adversarial point addition and deletion attacks by randomly adding and removing points in input data to design models that are robust to ID shifts. Additionally, Dong et al. [50] leveraged the relative positions of local features, and  Sun et al. [51] emphasized learning local features through self-supervision.

In contrast to ID shifts, OOD shifts occur when the test data distribution diverges significantly from the training data, often involving previously unseen classes or environments. Veeramacheneni and Valdenegro-Toro [52] established a benchmark for semantic segmentation utilizing Semantic3D, which consists of outdoor scenes, and S3DIS, comprising indoor scenes. Bhardwaj et al. [53] employed knowledge distillation for object classification, while  Riz et al. [54] applied online clustering and uncertainty quantification to generate prototypes for pseudo-labeling point clouds of previously unseen classes. The research on object detection [55,56,57], instance segmentation [58], and semantic segmentation [57] in outdoor environments for autonomous driving has focused on enhancing robustness against distributional shifts caused by previously unseen and unknown objects. The 3DOS benchmark [59] evaluates models, encompassing both in-domain scenarios (synthetic-to-synthetic and real-to-real) and cross-domain scenarios (synthetic-to-real). Research dedicated to OOD shifts using FSL for 3D point clouds remains limited. The BelHouse3D benchmark [60] examines FSL under such shifts in *indoor scene segmentation*, identifying inherent point cloud occlusion that alters the shapes and context of 3D household objects. Unlike prior works, our study focuses on investigating a wide range of shifts that are particularly encountered in railway environments.

### 2.2. Notation and Problem Setup

Assume a point cloud, $X=\{x_{1},x_{2},\cdots ,x_{n}\}$, which is an unordered set of *n* points in Euclidean space. Each point $x_{i}=\left(x_{i},y_{i},z_{i}\right)\in R^{3}$ is a vector representing the *i*-th point in three-dimensional space and corresponds to one of the *l* target labels, $C=\{c_{1},c_{2},\cdots ,c_{l}\}$. Let $f_{\theta }\left(\cdot \right)$ denote a deep neural network with learnable parameters θ. We train the model $f_{\theta }\left(\cdot \right)$ on a labeled training set, $D_{T}$, to evaluate its generalization capability on an evaluation (test) set, $D_{E}$. Conditioned on $D_{E}$, we formulate distributional shifts to represent the types of changes encountered by real-world railway monitoring systems as follows:

1. **ID shift:** $D_{E}$ is constructed by sampling $n_{E}$ point clouds, ${\left\{X_{i}\right\}}_{i=1}^{n_{E}}$ drawn i.i.d. from the same distribution as the training set, $D_{T}$, and then adding random noise, X,
  (1) $$D_{E}=\left\{X_{i}+X\right\}.$$
  X simulates sensor noise as represented by variations in object positions and orientations. We employ three types of noise in our transformations: jitter, mirroring, and rotation. Jitter adds Gaussian noise to the point cloud X:
  (2) $$X^{\prime }=X+clip\left(\sigma \cdot N\left(0,I\right),-b,b\right),$$
  where σ=0.01, b=0.05, and N(0,I) represent a standard normal distribution sampled for each point in X. Mirroring randomly reflects the point cloud along the x-axis and y-axis. Rotation rotates the point cloud around the z-axis by an angle sampled from a uniform distribution, angle∼Uniform(0,2π).
2. **In-domain OOD shift:** $D_{E}$ consists of samples from classes that were not included in the training phase, taking into account a dataset, *D*, which encompasses $C=\{c_{1},c_{2},\cdots ,c_{j},\cdots ,c_{l}\}$ classes. The training set, $D_{T}$, contains samples from *j* classes, referred to as base classes, denoted as $C_{base}=\left\{c_{1},c_{2},\cdots ,c_{j}\right\}$, where j<l. In contrast, $D_{E}$ includes samples from the remaining l−j classes, designated as novel classes, $C_{novel}=C\setminus C_{base}=\left\{c_{j+1},\cdots ,c_{l}\right\}.$ The base and novel classes are contextually distinct and mutually exclusive, ensuring that $C_{base}\cap C_{novel}=\emptyset$. This situation represents an *OOD shift within the same domain*, characterized by the condition $P\left(D_{T}|C_{base}\subset D\right)\neq P\left(D_{E}|C_{novel}\subset D\right)$. This shift is indicative of the emergence of new classes during upgrades to railway infrastructure.
3. **Cross-domain OOD shift:** $D_{E}$ is constructed by sampling test samples from a dataset, $D_{B}$, that is entirely distinct from $D_{A}$, which was utilized to create the training set, $D_{T}.$ The datasets, $D_{A}$ and $D_{B}$, are sourced from different distributions, resulting in an OOD shift due to domain differences, expressed as $P\left(D_{T}|D_{A}\right)\neq P\left(D_{E}|D_{B}\right).$ For simplicity, our experimental setup assumed that $D_{T}$ and $D_{E}$ share the same set of classes, $C=\{c_{1},c_{2},\cdots ,c_{l}\}$. This shift reflects variations arising from differences in railway environments across diverse geographical regions, such as Asia and Europe, or among various countries.

**Figure 1:**
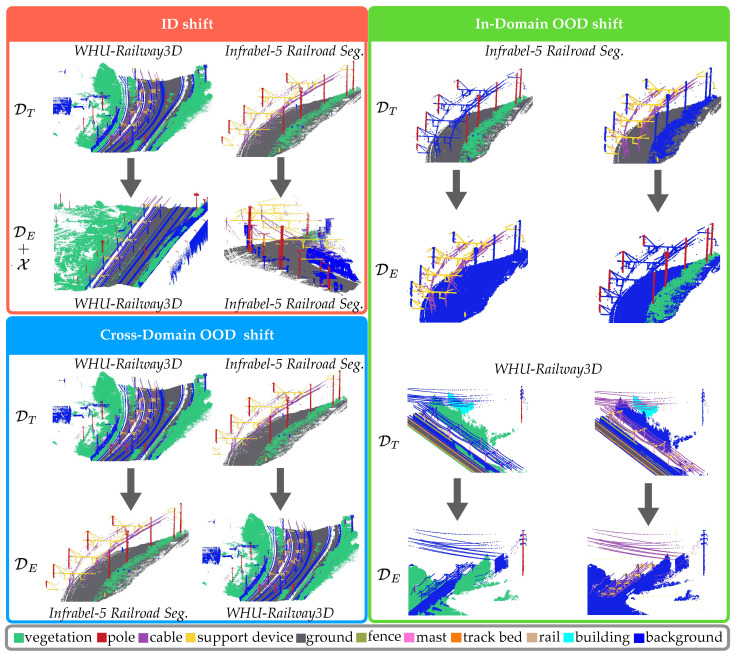
The experimental setup illustrates three types of distributional shifts: (i) ID shift (red box), (ii) in-domain OOD shift (green box), and (iii) cross-domain OOD shift (blue box) using the Infrabel-5 Railroad Segmentation dataset and the WHU-Railway3D dataset. Class labels are shown in the gray box. Rows represent the training ($D_{T}$) and evaluation ($D_{E}$) sets for each shift type.

Figure 1 presents an overview of the distributional shifts, as captured by the datasets used in our experimental setup.

## 3. Materials and Methods

### 3.1. Few-Shot Learning

FSL is closely related to the concept of generalization over test tasks, where a learned model is expected to adapt to new tasks, provided with minimal supervision at test time. This concept mimics the learning capabilities inherent in human intelligence. For instance, a child can identify a zebra in a zoo after seeing only a few pictures of zebras in a book, even without direct prior exposure to the animal. Theories from cognitive psychology suggest that humans and animals learn by capturing the regularities and commonalities among categories of objects, forming internal representations, defined as *prototypes* [61,62,63,64,65,66,67,68]. These prototypes are then used to categorize an unseen object as belonging to a particular category (or not).

We define FSL on 3D point cloud segmentation tasks for railway environments under distributional shifts to study a model’s generalization capability at test time. Each task samples *K* labeled examples and *q* test examples from an example space to constitute a pair of support–query sets (S,Q) for *N* chosen classes, known as an *N-way K-shot task*. FSL aims to train the model $f_{\theta }\left(\cdot \right)$ from Section 2.2 over the distribution of tasks, $P(T_{T})$, which samples (S,Q) pairs from $D_{T}$ during the meta-training phase. The goal is to generalize to a distribution of previously unseen tasks, $P(T_{E})$, consisting of (S,Q) pairs sampled from the test domain. Since the problem essentially becomes a transfer learning task under OOD shifts, we assume that minimal supervision is available through a few labeled samples from the test domain, which provide prior knowledge about the domain. These labeled examples are used to construct S for $P(T_{E})$, while Q comprises the test samples to be evaluated. This Q is effectively equivalent to $D_{E}$. Under this setting, we characterized the distributional shifts (see Section 2.2) as follows:

- **ID shift:** $P(T_{T})$ and $P(T_{E})$ are defined over *C* classes within a dataset *D*. $D_{E}$ is drawn i.i.d. from the same distribution as the $D_{T}$ with the inclusion of random noise. Consequently, $D_{E}$ and $D_{T}$ share the same underlying distribution, such that $P\left(T_{T}\right)\approx P\left(T_{E}\right)$.
- **In-domain OOD shift:** $P(T_{T})$ and $P(T_{E})$ are defined over $C_{base}$ and $C_{novel}$ classes within a dataset *D*, respectively. To ensure $P\left(T_{T}\right)\neq P\left(T_{E}\right),$$C_{base}$ and $C_{novel}$ are predefined rather than being chosen randomly.
- **Cross-domain OOD shift:** $P(T_{T})$ and $P(T_{E})$ are defined for *C* shared classes from two distinct datasets, $D_{A}$ and $D_{B}$, respectively. The difference in underlying distributions ensures that $P\left(T_{T}\right)\neq P\left(T_{E}\right)$.

To incorporate the prototypical view, we employ Prototypical Networks [69], a commonly used metric-based method for FSL. They utilize $f_{\theta }\left(\cdot \right)$ initialized with a pretrained weight to compute the feature representation $z_{i}$ for each point $x_{i}$ in X,

(3) $$z_{i}=f_{\theta }\left(x_{i}\right)\;\forall x_{i}\in X.$$

In an *N*-way *K*-shot task, the prototype for each foreground class $c_{i}=\left\{1,\cdots ,N\right\}$ is computed as the mean embedding of all points within that class given *K* examples from S. The background prototype is calculated as the mean embedding of points that do not belong to any of the *N* classes.

(4) $$\begin{array}{c} {\bar{c}}_{i}=\frac{1}{|K\times X_{c_{i}}|}\sum_{x_{i}\in X_{c_{i}}}f\left(x_{i}\right),\;\mathrm{and}\;{\bar{c}}_{i}^{bg}=\frac{1}{|K\times X_{c_{i}}|}\sum_{x_{i}\notin X_{c_{i}}}f\left(x_{i}\right). \end{array}$$

Given a query point cloud, X∈Q, the probability for each point $x_{q}\in X$ to belong to a class, $c_{i}\in \{1,\cdots ,N+1$} is obtained by applying a softmax over the distances to each class prototype:

(5) $$P\left({\hat{y}}_{q}=c_{i}|x_{q},\theta \right)=\frac{\exp(\int \left(f_{\theta }\left(x\right),\bar{c_{i}}\right))}{\sum_{c_{i}=1}^{N+1}\exp\left(\int \left(f_{\theta }\left(x\right),\bar{c_{i}}\right)\right)},$$

where N+1 represents the total number of classes, including *N* foreground classes and 1 background class, ${\hat{y}}_{q}$ is the predicted class label for point $x_{q}$, and ∫ denotes the similarity/distance function, which in our experiments is implemented as the cosine distance.

**Figure 2:**
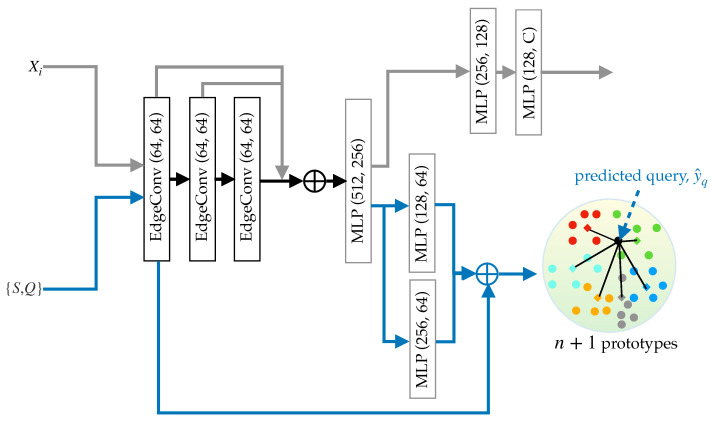
Overview of our pipeline. The network is trained on input point clouds ($X_{i}$) using batches from a subset of the WHU-Railway3D dataset in a supervised manner (top part, connected via gray arrows). The trained network is then utilized as a feature extractor for FSL given a pair of support–query (S,Q) sets (bottom part, connected via blue arrows). Specifically, we illustrate a 5-way task, producing five foreground prototypes (depicted in red, green, blue, yellow, and sky blue) and one background prototype (depicted in gray). The query feature is then classified to the nearest prototype based on cosine distance.

Figure 2 presents our pipeline for point cloud segmentation in railway monitoring, illustrating the pretraining process and FSL. We employ DGCNN [70], pretrained on a subset of WHU-Railway3D dataset, to extract support and query features using Equation (3). DGCNN constructs a local neighborhood graph for each point using *k*-Nearest Neighbors (*k*-NN) and applies a convolutional operation to the edges via EdgeConv layers to capture local geometric structures, crucial in railroad segmentation. The network aggregates features for each point by pooling the edge features from each subsequent EdgeConv layer. These aggregated features are then input into multi-layer perceptrons to produce global feature representations at different scales, which are combined to enhance the overall representation. The graph in DGCNN is dynamic as it is recomputed at every layer, enabling the network to adjust neighborhood relationships based on the learned features at each stage and facilitating learning complex and non-linear relationships within point clouds.

We conducted experiments using Transformer-based models, specifically Point Transformers v1, to be used as the pretrained network. Observations indicate that the model tends to overfit on small datasets like those used in our experiments. Furthermore, recent work [71] comparing DGCNN with Transformer-based models, particularly Convolutional Point Transformer (CpT), reports closely comparable performance. This finding further reinforces our decision to prioritize the computationally efficient DGCNN architecture.

### 3.2. Datasets

**Infrabel-5 Railroad Segmentation dataset.** The Infrabel-5 Railway Segmentation dataset consists of data from 8 railway tracks, each containing 8 million raw points, collected across various cities in Belgium. The data collection process is administered by Infrabel (a Belgian government-owned public limited company responsible for the installation, upgrading, and maintenance of railway infrastructure). The point clouds, represented by *x*, *y*, and *z* coordinates, are captured using a Z+F 9012 LiDAR-based MMS system. The raw data were shared with the authors under a confidentiality agreement and were also used in our earlier work [72]. The data were initially filtered by removing outliers using the Statistical Outlier Removal (SOR) filter within the CloudCompare software (available at https://www.danielgm.net/cc/, (accessed on 20 December 2024)), followed by manual annotation using the software’s built-in annotation tool. The filtered dataset contains approximately 4 million points per railway track, classified into 5 object classes: cable, cable holder (support device), pole, vegetation, and ground. Any remaining points are categorized as clutter. Although this dataset accurately represents the highly imbalanced class scenarios common in real-world railway environments, it does not include RGB information.

**WHU-Railway3D dataset.** The WHU-Railway3D dataset [73] is a publicly available collection of 3D point clouds, represented by *x*, *y*, and *z* coordinates, capturing railroad infrastructure across urban, rural, and plateau environments. An Optech Lynx MMS system captured 675 million points over 10.7 km in urban areas. In rural environments, a HiScan-Z MMS system captured 2775 million points across 10.6 km, while a rMMS system collected 362 million points over 10.4 km in plateau areas. The maximum valid range for urban data is 250 m with an outlier tolerance of 0.2%, and the average point density is 2000 points/m^2^. The rural and plateau data have a maximum valid range of 119 m with an outlier tolerance of 0.1%. The average point density is 9000 points/m^2^ in rural areas and 500 points/m^2^ in plateau regions. Across all environments, the point position accuracy is 5 cm. The dataset spans over 30 km with 3.9 billion points, manually annotated into 10 classes: rail, track bed, mast, support device, overhead line (cable), fence, pole, vegetation, building, and ground. Notably, the dataset lacks RGB information.

## 4. Experiments

Our experiments evaluated the generalization capability of the FSL model under distributional shifts formulated in Section 2.2 for 3D point cloud segmentation in railroads. The experimental setup for distributional shifts, including data splits and class configurations for training and evaluation, is presented in Table 1.

First, we assessed the model under a *no-shift scenario*, which replicates the ID shift scenario without introducing additional noise (X) to the evaluation set ($D_{E})$. This scenario serves as the baseline for comparison with distributional shifts. For ID shifts, the trained model is evaluated on $D_{E}$ for both datasets, incorporating jitter, mirroring, and rotation (Equation (1)).

For the in-domain OOD shift, the model was evaluated on two class-based configurations: {pole,vegetation} and {cable,support device} for both datasets, while the remaining classes were used during the training phase. For the cross-domain OOD shift, the model was trained on the WHU-Railway3D dataset and evaluated on the Infrabel-5 Railroad Segmentation dataset and vice versa. Although both datasets share the same classes—pole, vegetation, ground, cable, and support device—in our experimental setup, they significantly differ in terms of distributions. We do not include the clutter class from both datasets in FSL training.

### 4.1. Evaluation Metrics

**Performance metrics.** Three metrics, *mean Intersection over Union (**mIoU**)*, *Overall Accuracy (**OA**)*, and *Matthews Correlation Coefficient (**MCC**)*, are employed to assess the model’s performance. Given the four categories of the confusion matrix—true positive (TP), false positive (FP), true negative (TN), and false negative (FN)—these metrics are defined as follows:

- **mIoU:** *mIoU* measures how well each class is segmented by measuring the overlap between predicted and ground-truth points for each class.
  (6) $$mIoU=\frac{1}{N}\sum_{i=1}^{N}\frac{TP_{c}}{TP_{c}+FP_{c}+FN_{c}}.$$
  Note that we ignore the background class for our evaluation.
- **OA:** *OA* measures the ratio of correctly classified points over the total number of points, regardless of classes, thereby reflecting the overall correctness of the model’s predictions.
  (7) $$OA=\frac{\sum_{i=1}^{N+1}\left(TP_{i}+TN_{i}\right)}{\sum_{i=1}^{N+1}\left(TP_{i}+FP_{i}+TN_{i}+FN_{i}\right)}.$$
- **MCC:** In a class imbalance scenario, the *OA* can present overly optimistic model performance evaluations. In contrast, *MCC* provides a more equitable assessment of classification models. It yields a high score only when the predictions are accurate across all four categories of the confusion matrix—TP, FP, TN, and FN—while also considering the relative sizes of both positive and negative classes within the dataset. This makes the *MCC* a robust metric for performance evaluation, particularly in imbalanced datasets.
  (8) $$MCC=\frac{\sum_{i=1}^{N+1}\left(TP_{c}\times TN_{i}\right)-\left(FP_{i}\times FN_{i}\right)}{\sqrt{\prod_{i=1}^{N+1}\left(TP_{i}+FP_{i}\right)\left(TP_{i}+FN_{i}\right)\left(TN_{i}+FN_{i}\right)\left(TN_{i}+FN_{i}\right)}}$$
  This metric ranges from −1, indicating total disagreement, to 1, representing perfect prediction.

**Uncertainty metric.** Entropy quantifies the model’s performance in terms of probabilistic predictions, providing insights into the model’s uncertainty in its predictions. Given a point, entropy is defined as,

(9) $$H\left(p\right)=-\sum_{i=1}^{N+1}p_{i}\log\left(p_{i}\right),$$

where $p_{i}$ denotes the probability that a point belongs to class, $c_{i}\in N+1$. We report the *mean entropy (mH)*, which calculates the average entropy across all points in the evaluation set. A lower mean entropy value indicates higher model confidence in its predictions.

### 4.2. Implementation Details

During the pretraining phase, the model is trained for 200 epochs on the WHU-Railway3D dataset using the Adam optimizer with a learning rate of $1\times 10^{-4}$ and L2 regularization of $1\times 10^{-5}$. The learning rate is reduced by a factor of 0.5 after 100 epochs. A batch size of 16 is used. For episodic training in FSL, 40K episodic tasks were sampled from $D_{T}$. The Adam optimizer was employed with a learning rate of $1\times 10^{-4}$, which was halved every 5000 episodes. The trained model was evaluated on 1500 test tasks sampled from $D_{E}$. An NVIDIA Tesla V100 GPU with 32 GB of memory was used for training. The code was implemented in PyTorch and, along with the pretrained model, is publicly available at https://gitlab.kuleuven.be/eavise/point-clouds/dist_shift_railroads_fsl.git, (accessed on 1 February 2025).

To efficiently utilize limited computational resources, we adopted the data preprocessing strategy from PointNet, which employs a 3D sliding cubic window of size x×y×z to subdivide a large point cloud into smaller cubic blocks. This sampling approach has also been utilized in PointNet++, DGCNN, and many others. In our case, x=1m, y=1m, and *z* equals the maximum height (in meters) recorded in the point cloud. This process captures points within 1m×1m cubic blocks, from which we randomly sample 2048 points as input to the network. We also conducted preliminary experiments using 1024 and 4096 points. Using 2048 points enabled the model to capture more representative features compared to 1024. However, increasing the number of points to 4096 did not yield significant performance benefits. Thus, 2048 points provided a favorable trade-off between computational efficiency and model performance.

## 5. Experimental Results and Analysis

We conducted a comprehensive evaluation of our model across four scenarios: no-shift, ID shift, in-domain OOD shift, and cross-domain OOD shift, with no-shift serving as the baseline. The model performance is quantified using the following performance metrics: *mIoU*, *OA*, and *MCC*. Additionally, the model’s predictive uncertainty is assessed using *mH*. The average performance over 1500 test tasks is reported as the experimental results, implementing one-way twenty-shot tasks (i.e., N=1,K=20) for no-shift and ID-shift. For in-domain OOD shift and cross-domain OOD shift scenarios, the results are reported for one-way (i.e., N=1) tasks with five, ten, and twenty shots (i.e., K={5,10,20}). Additionally, in the cross-domain OOD shift scenario, the results are also reported for a five-way task setting (i.e., N=5) with five, ten, and twenty shots. A one-way task corresponds to a binary segmentation problem involving a single foreground class, while a five-way task represents a multi-class segmentation problem involving five foreground classes.

### 5.1. Quantitative Results

Table 2 presents the experimental results for the ID shift using the Infrabel-5 Railroad Segmentation dataset and the WHU-Railway3D dataset compared against the baselines under the no-shift scenario. The results indicate that the baselines achieved the highest performance and the lowest predictive uncertainty, with mIoU values of 89.1% and 83% and OA values of 92.4% and 89% for the Infrabel-5 Railroad Segmentation dataset and the WHU-Railway3D dataset, respectively. Under the ID shift scenario, with the application of jitter, mirroring, and rotation, the model’s performance remained comparable to the baselines, deviating by ~1% across all the metrics. These findings emphasize the strong generalization capability of FSL under minimal variations in evaluation conditions.

Table 3 and Table 4 summarize the experimental results for the *in-domain OOD shift* using one-way tasks. Specifically, Table 3 reports the results for two evaluation settings with previously unseen classes from the Infrabel-5 Railroad Segmentation dataset: $C_{novel}$ = {pole, vegetation} and $C_{novel}$ = {cable, support device}. In the $C_{novel}$ = {pole, vegetation} setting, the model achieved its best performance on twenty-shot tasks with 64.2% mIoU and 74.4% OA, representing ~7.5% mIoU and ~5.5% OA improvements over the second-best performance in ten-shot tasks. Conversely, in the $C_{novel}$ = {cable, support device} setting, the model attained comparable performance across five- and twenty-shot tasks, with the highest performance observed in ten-shot tasks, ~1% better than in both other settings. The lowest predictive uncertainty was recorded in twenty-shot tasks for the $C_{novel}$ = {pole, vegetation} and in five-shot tasks for $C_{novel}$ = {cable, support device}.

Table 4 presents the results for the same evaluation settings as Table 3 but applied to the WHU-Railway3D dataset. In the $C_{novel}$ = {pole, vegetation} setting, the model achieved its best performance on twenty-shot tasks with 76% mIoU and 78.4% OA, comparable to the results from ten- and twenty-shot tasks. For $C_{novel}$ = {cable, support device}, the highest performance was achieved in twenty-shot tasks with 59.1% mIoU and 83.9% OA, showing ~2.5% mIoU improvement over the second-best result in ten-shot tasks. The lowest predictive uncertainty occurred in five-shot tasks for the first setting and in ten-shot tasks for the second.

Table 5 and Table 6 provide the experimental results for the *cross-domain OOD shift*, evaluated on the Infrabel-5 Railroad Segmentation dataset and the WHU-Railway3D dataset, respectively. In Table 5, the model achieved its best performance for the one-way setting in twenty-shot tasks, with 77.5% mIoU and 84.8% OA, comparable to the second-best performance in ten-shot tasks. The lowest predictive uncertainty is observed in the ten-shot tasks. For the five-way setting, the model attained its highest performance in twenty-shot tasks, achieving 50% mIoU and 70.6% OA, with ~3.2% improvements in mIoU over ten-shot tasks (second best) and ~4% OA improvements compared to five-shot tasks. The twenty-shot task setting generates the lowest predictive uncertainty.

In Table 6, the model achieved its best performance for the one-way setting in ten-shot tasks, with 71.7% mIoU and 82.4% OA, comparable to the results (<1%) for five-shot and twenty-shot tasks. The lowest predictive uncertainty is observed under the same shot setting. For the five-way setting, the model performed best in twenty-shot tasks, with 48% mIoU and 62.4% OA, showing ~2.5% mIoU and ~1.6% OA improvements compared to the second-best results achieved in ten-shot tasks. The lowest predictive uncertainty was observed in the twenty-shot tasks.

### 5.2. Qualitative Results

**Figure 3:**
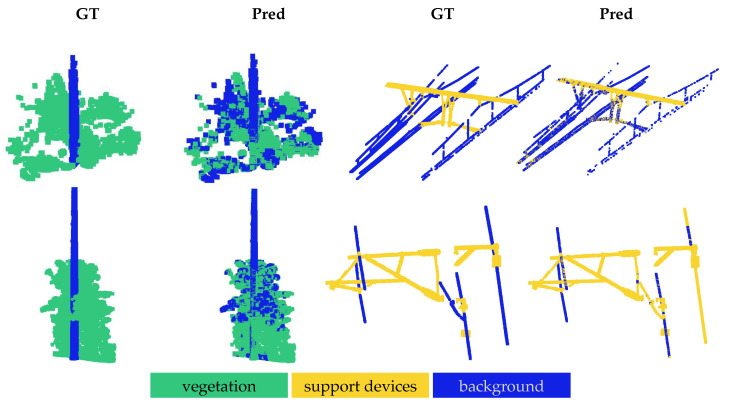
Qualitative results for in-domain OOD shift. The first row shows results for the Infrabel-5 Segmentation dataset, while the second row corresponds to the WHU-Railway3D dataset. In each row, previously unseen foreground classes—vegetation and support device—are segmented from the background, illustrating the two evaluation settings.

**Figure 4:**
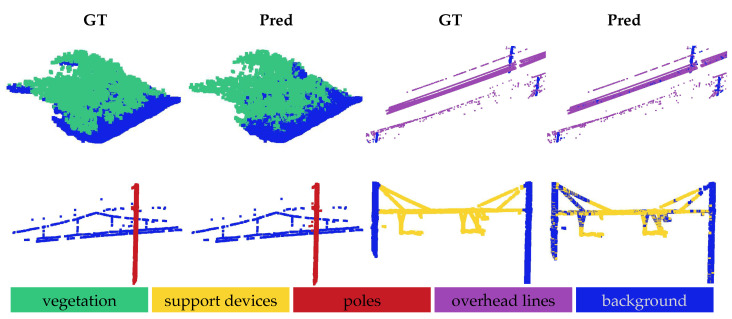
Qualitative results under cross-domain OOD shift, evaluated on previously unseen Infrabel-5 Railroad Segmentation dataset. Results are shown for the 1-way 5-shot setting, comparing each model prediction (Pred) to the ground truth (GT). From left to right in two rows, segmentation of four foreground object classes is illustrated: vegetation, overhead line, pole, and support device.

**Figure 5:**
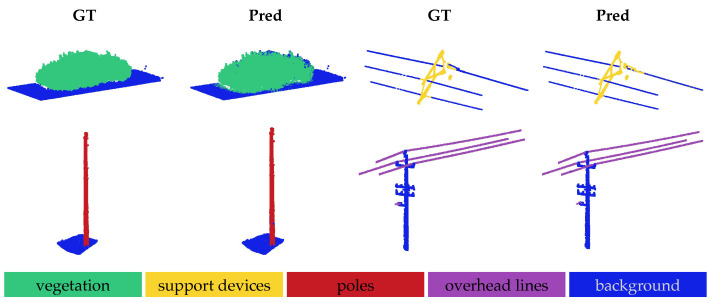
Qualitative results under cross-domain OOD shift, evaluated on previously unseen WHU-Railway3D dataset. Results are shown for the 1-way 5-shot setting, comparing each model prediction (Pred) to the ground truth (GT). The first row illustrates two foreground object classes, vegetation and overhead line (from left to right), and the second row shows poles and support devices (from left to right).

The qualitative results for the OOD shifts are illustrated in Figure 3, Figure 4 and Figure 5. In all the figures, the model was evaluated via a one-way five-shot setting, with smaller regions extracted from larger railroad areas for clear visualization. The ground truth is displayed in the ‘GT’ columns, while the model predictions are shown in the corresponding ‘Pred’ columns.

Figure 3 illustrates the results for the in-domain OOD shift, showing the results for the Infrabel-5 Segmentation dataset in the first row and the WHU-Railway3D dataset in the second row. In the first two columns of each row, vegetation is segmented by the model that was trained on ground, cable, and support devices. In the last two columns, support devices are segmented using the model that was trained on ground, vegetation, and poles. The results indicate better segmentation of vegetation from poles in the WHU-Railway3D dataset compared to the Infrabel-5 dataset. Conversely, the model performed better at segmenting support devices from cables in the Infrabel-5 dataset, while some cables were misclassified as support devices in the WHU-Railway3D dataset. Overall, the figure highlights the model’s strong generalization to novel railway infrastructure classes despite receiving minimal supervision, with only five labeled support examples.

Figure 4 and Figure 5 present the qualitative results for the cross-domain OOD shift, evaluated on the Infrabel-5 Segmentation dataset and the WHU-Railway3D dataset, respectively, with the other dataset used for training. These datasets exhibit notable structural differences: the vegetation and poles are taller in the WHU-Railway3D dataset, while the support devices vary significantly in form. Both figures demonstrate that the model generalizes well to previously unseen distributions, although some challenges remain in segmenting vegetation (row 1, columns 1–2) and distinguishing support devices from cables (row 2, columns 3–4). Nonetheless, the model accurately segments poles and cables when presented against distinct backgrounds.

### 5.3. Ablation Study

**Fine-tuning.** Fine-tuning is often used to adapt a model to the test distribution when it significantly differs from the training distribution. We fine-tuned the pretrained model on the Infrabel-5 Railroad Segmentation dataset. The last two layers of the pretrained model were fine-tuned on the WHU-Railway3D dataset to better align the model with our selected evaluation classes. We employed the Adam optimizer with a learning rate of $1\times 10^{-4}$ and weight decay of $1\times 10^{-5}$. Training and evaluation follow the same data splits as the ID shift (Table 1), with a batch size of 16. The training is conducted for 30 epochs.

Table 7 presents the fine-tuning results for our segmentation task. The results show that fine-tuning achieves more than 60% mIoU in both datasets, achieving 92.6% OA for the WHU-Railway3D dataset, which is 10% higher than the OA value for the Infrabel-5 Railroad Segmentation dataset.

## 6. Discussion

This work discusses the generalization of FSL under inevitable distributional shifts encountered in real-world railroad monitoring, arising from sensor noise (ID shift), infrastructure upgrades (in-domain OOD shift), and environmental variations across geographical regions (cross-domain OOD shift). The baseline performances achieved under the no-shift condition (Table 2), representing optimal similarity to the training conditions, provide an upper bound for comparison. The results for ID shift (Table 2), compared to the baselines, demonstrate minimal impact on model performance, with deviations of ~1%, highlighting FSL’s strong generalization ability under minor variations in evaluation conditions.

**Figure 6:**
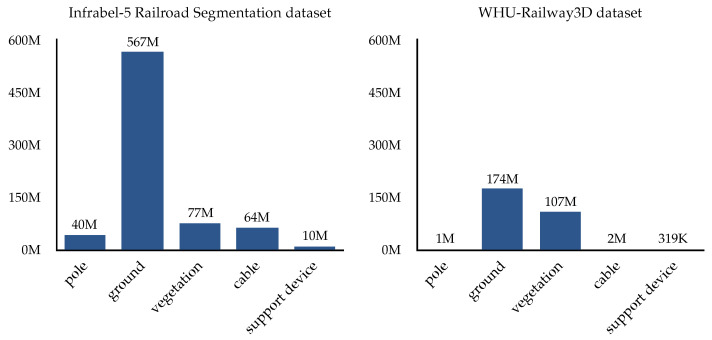
Distribution of points per class, highlighting the highly imbalanced nature of our datasets: the Infrabel-5 Segmentation dataset (on the **left**) and the WHU-Railway3D dataset (on the **right**).

The in-domain OOD shift, which evaluated the model on novel, previously unseen classes from the same dataset as the training set, indicates a performance drop. For the Infrabel-5 Railroad Segmentation dataset, the {pole, vegetation} setting experienced a ~24.9% decrease in mIoU and 18.1% reduction in OA compared to the baseline (Table 3 vs. Table 2). In contrast, the {cable, support device} setting exhibited a 1.45% decline in mIoU with a comparable OA. For the WHU-Railway3D dataset (Table 4 vs. Table 2), the {pole, vegetation} setting demonstrates a ~7.4% mIoU and ~10.7% OA decrease, while the {cable, support device} setting shows a ~23.9% mIoU and ~5.1% OA decline. These performance differences across the datasets (Table 3 vs. Table 4) for the two evaluation settings are likely attributable to class imbalances between the datasets. Vegetation has higher representation in the WHU-Railway3D dataset, whereas cable and support device classes are more prevalent in the Infrabel-5 Segmentation dataset (Figure 6).

The cross-domain OOD shift with one-way tasks resulted in performance decreases of ~11.3–11.6% in mIoU and ~6.6–7.6% in OA when compared to the baselines (Table 5 and Table 6 vs. Table 2) for both datasets. Specifically, the mIoU and OA decreased by 11.6% and 7.65% on the Infrabel-5 Railroad Segmentation dataset and by 11.3% and 6.6% on the WHU-Railway3D dataset.

*N-way K-shot* tasks were examined for different *N* and *K* configurations. Under cross-domain OOD shifts, increasing N=1 to N=5 led to performance drops of 27.5% mIoU and ~14.2% OA on the Infrabel-5 Railroad Segmentation dataset, and ~23.7% mIoU and ~19.9% OA on the WHU-Railway3D dataset. These results align with existing FSL research, where the increased complexity of multi-class segmentation (N=5) tasks contributes to the observed performance disparity. Increasing *K* to 20 notably enhanced the model performance in some settings. For instance, in the in-domain OOD shift for the {pole, vegetation} setting on the Infrabel-5 Railroad Segmentation dataset, the mIoU and OA improved by ~12.1% and ~10%, respectively, compared to K=5 (Table 3). Similarly, for the {cable, support device} setting on the WHU-Railway3D dataset, the performance increased by ~2.5% in mIoU and ~1.6% in OA (Table 4). For cross-domain OOD shift using one-way tasks, improvements of over 4% in both mIoU (4.43) and OA (4.16) were observed compared to K=5 for the Infrabel-5 Segmentation dataset. Similar behavior was observed in the five-way tasks, where increasing *K* resulted in ~4-4.4% gains in both mIoU and OA across both the Infrabel-5 Segmentation dataset and WHU-Railway3D dataset (Table 5 and Table 6). However, in other scenarios, the number of support examples had a minimal effect on performance. While increasing *K* boosted the results, it occasionally introduced noise in some cases, leading to slight reductions in model performance.

This study underscores the importance of evaluating model performance using the *MCC* metric, which is particularly useful for identifying trivial majority classifiers. An *MCC* value of 0 indicates a model that predicts the majority class irrespective of input features [74]. Given the highly class-imbalanced nature of our datasets, *MCC* offers a more realistic assessment of model performance as it penalizes errors in minority classes more effectively than *OA*. The baseline results (Table 2) exhibit 82% agreement with the ground truth for the Infrabel-5 Segmentation dataset and 77% for the WHU-Railway3D dataset, while the corresponding OA values are higher, i.e., 92% and 89%, respectively. Similar trends are observed across all the experiments under distributional shifts. In those experiments involving one-way tasks, *MCC* consistently exceeded 60%, except in the in-domain OOD shift for the {pole, vegetation} setting, where it decreased to 47% for the Infrabel-5 Segmentation dataset and 39% for the WHU-Railway3D dataset, respectively.

The comparison between the FSL baselines under no-shift (Table 2) and fine-tuning (Table 7) shows that FSL achieved an 18.1% improvement in mIoU on the Infrabel-5 Railroad Segmentation dataset and a 21.3% improvement on the WHU-Railway3D dataset. Under cross-domain OOD shift with the one-way task setting (Table 5 and Table 6 vs. Table 7), FSL achieved 17.5% and 9.9% mIoU improvements on the Infrabel-5 Railroad Segmentation dataset and WHU-Railway3D dataset, respectively. However, such cross-domain OOD generalization with limited data is not inherent to fine-tuning as fine-tuning relies on the i.i.d. assumption and is evaluated on a test set drawn from the same distribution as the training data. Compared to fine-tuning, the FSL baselines (under no-shift) yielded a higher *mH* value on the WHU-Railway3D dataset. However, on the Infrabel-5 Railroad Segmentation dataset, *mH* was lower, suggesting that the model exhibits greater confidence when trained on a smaller dataset with FSL. Under cross-domain OOD shift, FSL resulted in higher *mH* values, reflecting increased uncertainty in unseen environments. Nevertheless, the model remains capable of generalizing beyond the i.i.d. assumption—an aspect where fine-tuning falls short. These findings further highlight the importance of incorporating predictive uncertainty measures, such as entropy, in the evaluation of FSL models.

**Figure 7:**
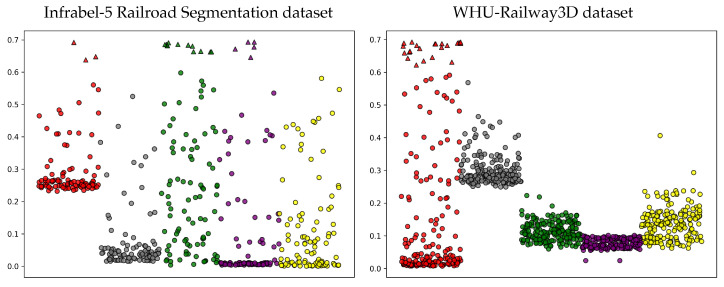
Sample-wise entropy plot for the first 100 samples of each class in the Infrabel-5 Railroad Segmentation dataset (**left**) and the WHU-Railway3D dataset (**right**) under cross-domain OOD shifts. Classes, poles, ground, vegetation, overhead lines, and support devices are represented in red, gray, green, purple, and yellow, respectively. Highly uncertain samples are represented as triangles.

The entropy measure provides insights into the reliability of our model’s predictions by quantifying uncertainty, which is crucial for real-world applications such as railroad monitoring, where distributional shifts are inevitable. Since test data often lack ground truth for verification, quantifying predictive outcomes through entropy can support decision-making. When the model exhibits low confidence in its predictions, highly uncertain samples can be referred to human experts for further review. Figure 7 illustrates this for the Infrabel-5 Railroad Segmentation and WHU-Railway3D datasets under cross-domain OOD settings, highlighting highly uncertain samples for poles (red), ground (gray), vegetation (green), cables (purple), and support devices (yellow). For instance, three highly uncertain pole samples (red) in the Infrabel-5 Railroad Segmentation dataset could be flagged for human evaluation in critical decision-making scenarios. Moreover, in domain adaptation with FSL, *mH* provides essential insights: a high *mH* value indicates low model confidence under a new distribution, aiding in decisions such as model rejection, recalibration, or further fine-tuning.

## 7. Challenges and Future Prospects

Our work currently has certain limitations, primarily linked to the availability of only x,y,z coordinates as features in our datasets. Segmenting vegetation from the ground solely based on *x*, *y*, *z* coordinates presents significant challenges as the most critical features often rely on height (*z* coordinates). In complex environments like railways, the height of the ground plane can vary, particularly between the track bed and its surroundings. Incorporating additional information, such as RGB color data, could enhance the model’s ability to learn more robust features. Furthermore, the development of more balanced training datasets is crucial for improving the effectiveness and generalization of machine learning models in railway monitoring. In the future, model calibration or active learning can be explored in conjunction with FSL for models under such distributional shifts.

## 8. Conclusions

Machine learning models are often evaluated under the i.i.d. assumption for training and test sets. However, this assumption does not hold true in dynamic real-world applications, particularly in complex railroad environments. In this work, we formulated distributional shifts that arise in railway environments due to sensor noise, infrastructure upgrades, or environmental variations across regions. We explored FSL for railway monitoring via the segmentation of 3D point clouds and evaluated its generalization capability under distributional shifts with minimal supervision at test time. We presented an extensive evaluation of the model performance, including an analysis and discussion, and assessed the model’s predictive confidence, emphasizing the crucial role of such evaluation in developing reliable models for real-world applications.

## Figures and Tables

**Table 1:** Dataset settings and class configurations used in our experiments on distributional shifts. The experiments utilize the Infrabel-5 Railroad Segmentation (Infrabel-5) dataset from Belgium (BEL) and the WHU-Railway3D (WHU) dataset from China (CHN).

Shift	Exp.	$D_{T}$ and $D_{E}$		Location
ID shift	i.	$D_{T}=$	Infrabel-5, area: 6, 7, 8	BEL
		$D_{E}=$	Infrabel-5, area: 5 (+X)	BEL
	ii.	$D_{T}=$	WHU, area: L5–L7, L10–L15, L17–L20	CHN
		$D_{E}=$	WHU, area: L14, L16 (+X)	CHN
In-domain	i.	$D_{T}=$	Infrabel-5, $C_{base}$: ground, pole, vegetation	BEL
OOD shift		$D_{E}=$	Infrabel-5, $C_{novel}$: cable, support device	BEL
	ii.	$D_{T}=$	Infrabel-5, $C_{base}$: ground, support device, pole	BEL
		$D_{E}=$	Infrabel-5, $C_{novel}$: pole, vegetation	BEL
	iii.	$D_{T}=$	WHU, $C_{base}$: ground, rail, track bed, mast	CHN
			fence, pole, vegetation, building	
		$D_{E}=$	WHU, $C_{novel}$: cable, support device	CHN
	iv.	$D_{T}=$	WHU, $C_{base}$: ground, rail, track bed, mast	CHN
			fence, cable, support device, building	
		$D_{E}=$	WHU, $C_{novel}$: pole, vegetation	CHN
Cross-domain	i.	$D_{T}=$	Infrabel-5	BEL
OOD shift		$D_{E}=$	WHU	CHN
	ii.	$D_{T}=$	WHU	CHN
		$D_{E}=$	Infrabel-5	BEL

**Table 2:** Quantitative results under ID shift, where X = {jitter, mirroring, rotation} applied to evaluation set. The first rows for both datasets with no X (under no-shift scenario) serve as the baselines. Results are reported for 1-way 20-shot tasks. The best performance metrics are shown in bold, and the second-best are underlined. Only the model with the lowest predictive uncertainty is highlighted in bold.

Evaluation Set	X	*mIoU*	*OA*	*MCC*	mH	
Infrabel-5	–	**89.10**	**92.48**	**0.8250**	**0.0938**	⇒ baseline
Infrabel-5	jitter	89.07	92.47	0.8247	0.0951	
Infrabel-5	mirroring	89.02	92.43	0.8237	0.0952	
Infrabel-5	rotation	89.01	92.42	0.8236	**0.0938**	
WHU	–	**83.04**	**89.02**	**0.7714**	**0.3254**	⇒ baseline
WHU	jitter	82.89	88.86	0.7682	0.3261	
WHU	mirroring	82.95	88.97	0.7701	0.3255	
WHU	rotation	82.94	88.96	0.7700	0.3256	

**Table 3:** Quantitative results for the in-domain OOD shift scenario based on two evaluation settings using the Infrabel-5 Railroad Segmentation dataset. Results are reported for N=1. The highest performance metrics and the lowest predictive uncertainty for each setting are highlighted in bold. Higher values indicate better performance for mIoU, OA, and MCC (↑), while lower values indicate better performance for mH (↓).

	{pole, vegetation}				{cable, support device}		
Metric	K=5	K=10	K=20		K=5	K=10	K=20
mIoU ↑	52.01	56.70	**64.17**		86.56	**87.65**	86.08
OA ↑	64.32	68.92	**74.38**		91.32	**92.46**	91.18
MCC ↑	0.2804	0.3786	**0.4708**		0.8091	**0.8292**	0.8086
mH ↓	0.4914	0.5002	**0.4754**		**0.0938**	0.1149	0.1007

**Table 4:** Quantitative results for the in-domain OOD shift scenario based on two evaluation settings using the WHU-Railway3D dataset. Results are reported for N=1. The highest performance metrics and the lowest predictive uncertainty for each setting are highlighted in bold.

	{pole, vegetation}				{cable, support device}		
Metric	K=5	K=10	K=20		K=5	K=10	K=20
mIoU ↑	75.31	75.41	**76.03**		56.46	56.61	**59.12**
OA ↑	77.91	77.63	**78.46**		82.26	82.38	**83.87**
MCC ↑	0.3828	0.3520	**0.3841**		0.6441	0.6449	**0.6673**
mH ↓	**0.3315**	0.4803	0.6312		0.4809	**0.4716**	0.5321

**Table 5:** Quantitative results under cross-domain OOD shift, with the WHU-Railway3D dataset as the training set and the Infrabel-5 Railroad Segmentation dataset as the evaluation set. The best performance metrics and lowest predictive uncertainty for N={1,5} are shown in bold.

	N=1				N=5		
Metric	K=5	K=10	K=20		K=5	K=10	K=20
mIoU ↑	73.05	76.65	**77.48**		46.72	46.02	**49.98**
OA ↑	80.67	83.80	**84.83**		66.49	66.43	**70.58**
MCC ↑	0.5867	0.6523	**0.6764**		0.5534	0.5515	**0.6022**
mH ↓	0.3437	**0.2108**	0.2358		0.7589	0.7784	**0.7418**

**Table 6:** Quantitative results under cross-domain OOD shift, with the Infrabel-5 Railroad Segmentation dataset as the training set and the WHU-Railway3D dataset as the evaluation set. The best performance metrics and lowest predictive uncertainty for N={1,5} are shown in bold.

	N=1				N=5		
Metric	K=5	K=10	K=20		K=5	K=10	K=20
mIoU ↑	70.94	**71.73**	70.29		43.58	45.48	**48.02**
OA ↑	81.75	**82.41**	81.37		58.43	60.83	**62.46**
MCC ↑	0.6227	**0.6371**	0.6153		0.4813	0.5123	**0.5367**
mH ↓	0.2385	**0.2173**	0.2901		0.7798	0.7278	**0.5514**

**Table 7:** Quantitative results on WHU-Railway3D dataset and Infrabel-5 Railroad Segmentation dataset using fully supervised setting.

Evaluation Set	*mIoU*	*OA*	*MCC*	mH
Infrabel-5	60.94	82.37	0.6864	0.1261
WHU	61.77	92.66	0.8854	0.1709

## Data Availability

The WHU-Railway3D dataset is publicly available from ‘WHU-Railway3D: A Diverse Dataset and Benchmark for Railway Point Cloud Semantic Segmentation’ at https://github.com/WHU-USI3DV/WHU-Railway3D/, (accessed on 5 January 2025). However, access to the Infrabel-5 dataset is restricted. This dataset was obtained from Infrabel, and access must be requested directly from them.
